# Family planning exemplar country selection methodology: time lag and trends analysis

**DOI:** 10.1136/bmjgh-2024-018771

**Published:** 2026-06-09

**Authors:** Zahid Ali Memon, Abeer Mian, Ira Martopullo, Narjis Fatima Hussain, Zeba Sathar, Ali Mohammad Mir, Michael Mbizvo, Marleen Temmerman, Jen Kidwell Drake, Zulfiqar A Bhutta

**Affiliations:** 1 Community Health Sciences, The Aga Khan University, Karachi, Pakistan; 2 Gates Ventures LLC, Seattle, Washington, USA; 3 Centre for Global Child Health, The Hospital for Sick Children, Toronto, Ontario, Canada; 4 Population Council Pakistan Office, Islamabad, Pakistan; 5 Population Council Zambia, Lusaka, Zambia; 6 Centre of Excellence in Women and Child Health, The Aga Khan University Hospital Nairobi, Nairobi, Kenya

**Keywords:** Global Health, Public Health

## Abstract

**Introduction:**

While many countries achieved notable gains in contraceptive use levels after the 1994 International Conference on Population and Development which redefined family planning (FP) as a human right, there was a long period of 15 years when contraceptive use levels did not receive due attention. It was only later that its subsequent inclusion in the Sustainable Development Goals and the 2012 London Summit on FP renewed global commitment and a focus towards FP. This paper aims to select countries that identify as ‘exemplar’ low-middle-income countries that have achieved exceptional progress in FP. Examining these outliers can uncover actionable drivers of success to accelerate progress in other countries that have not achieved as much success and are still experiencing stagnation in contraceptive prevalence rates.

**Methods:**

We selected exemplar countries based on exceptional performance across the outcome indicators of modern contraceptive prevalence and demand satisfied for modern methods, using a recent time frame post 2000 when focus on improving FP returned to the field. A selection approach was applied across various time periods to assess change in modern contraceptive prevalence rates (mCPR) and demand satisfied as the dependent variables. The Human Development Index for 2015 was used as a predictor of contraceptive use on a pooled sample of 130 countries.

**Results:**

The countries, defined by our criteria of residuals falling much higher than expected levels of predicted mCPR at a high level of significance, fell in the African continent. The countries selected in the first round were Senegal, Malawi, Kenya based on deviation from the predicted pattern at 5% significance level. In the second round, Sierra Leone from the African region, Lao People's Democratic Republic from Asia and Bolivia from Latin America, were added, on relaxing the level of significance, but still using the same approach. This was to have comparators in different regions.

**Conclusions:**

Our methodology, using a time lag analysis to build in an element of causation, offers a novel approach to selecting exemplar countries in family planning, based on the Human Development Index as a control metric.

**WHAT IS ALREADY KNOWN ON THIS TOPIC**

- Despite significant progress in expanding access to contraception since the 1990s, low-income and low-middle-income countries continued to face a substantial unmet need for family planning (FP). In 2012, over 200 million women in these countries expressed a desire to space or limit their pregnancies, but many do not use effective modern contraceptive methods. This is despite the proven safety and efficacy of these methods when used correctly. To address this challenge, the London Summit began the international effort to step up FP programmes.

**WHAT THIS STUDY ADDS**

- We aimed to develop objective metrics and comprehensive criteria for identifying positive outlier countries that respond to the renewed emphasis on FP and outperformed expected progress relative to secular trends on key FP outcomes. The primary outcome indicators analysed were (1) modern contraceptive prevalence rate and (2) demand satisfied for modern methods of contraception among women of reproductive age (15–49 years). Analysis over specified time periods was compared with changes in these two outcome indicators to the Human Development Index (HDI) in 2015, using a time lag analysis to assess the impact of HDI on contraceptive use and demand. Residuals were calculated using the level of significance to assess if the residual (Observed-Expected) for a country was significant at the 5% level of significance to highlight specific countries that performed significantly better from model predictions or expected trends based on earlier HDI metrics. The Exemplar countries for 2010–2020 fell mainly in Africa and were selected for more in-depth analysis. Additionally, using the same approach, but lowering the level of significance to 30% we added Bolivia and Lao People’s Democratic Republic to ensure broader geographical representation.

**HOW THIS STUDY MIGHT AFFECT RESEARCH, PRACTICE OR POLICY**

- This study employs a novel methodology for selecting exemplar countries in FP, characterised by objective metrics and criteria at the subregion and regional level. Deeper dives through mixed methods into these selected countries will provide relevant, applicable and robust evidence of impactful FP strategies and drivers of success to replicate at scale within peer countries in their respective regions.

## Introduction

 The 1994 International Conference on Population and Development (ICPD) marked an important shift in global reproductive health discourse. Adopted by 179 governments in Cairo, Egypt, the Conference redefined family planning (FP) through the lens of reproductive health and rights, laying the groundwork for future efforts. However, subsequent years witnessed inconsistent international funding for FP programmes (FPPs), correlating with fluctuations in fertility rates. The exclusion of FP from the initial Millennium Development Goals (MDGs) further underscored the global community’s wavering commitment to FP as a priority within reproductive health.

The London Summit on Family Planning in 2012, led by the Department for International Development, and the Bill & Melinda Gates Foundation and the UK Government, rekindled global interest and investment in FP, setting ambitious targets to expand access and improve quality of care. By focusing on mobilising resources and leadership around voluntary FP, the Summit aimed to add 120 million adolescent girls and women as users of modern contraceptives by the year 2020.^1^,^3^

FP is well known to have many societal benefits including reduction of maternal and infant mortality, improved economic development through increased women’s participation in the labour force, and more sustainable use of resources due to reduced population growth.^4^,^7^ However, many women do not practise FP despite their desire to delay or avoid childbearing.^4^ The United Nations Population Fund reports that globally, in 2022, 48.7% of all women of reproductive age (15–49 years) used a method of FP and 44.3% used a modern method of contraception.^8^ Among married women, 44.3% used any method; among them, 57% used modern methods for FP.^8^ Importantly, post-London Summit, the use of modern contraceptives significantly increased between 2012 and 2015 in some countries owing to improved policies and service strengthening at the national level. Consequently, the fertility rate also declined from 2.9 to 2.3 live births per woman from 1994 to 2021.^8^

Despite renewed momentum since 2012, FP investments and service access fall short of need in most low-resource settings.^9^ Current modern contraceptive methods are known to be both safe and effective when used as directed. Modern contraceptive methods, including pills, intrauterine devices (IUDs), condoms and implants, are designed to reliably prevent pregnancy through various physiological or barrier mechanisms. In contrast, traditional methods rely on behavioural practices or natural body observations (eg, rhythm method, withdrawal, exclusive breastfeeding), requiring strict adherence and generally exhibiting lower effectiveness rates due to their reliance on user behaviour and biological variability.^10^ A substantial number—over 200 million women in low-middle-income countries—express a desire to regulate spacing or limit the number of their children.^11^ Yet, these women either do not currently use these effective methods or rely on traditional contraceptive approaches. Even with greater contraceptive adoption, almost 40% of women using contraceptives discontinue use within the first year due to dissatisfaction.^11^

Notwithstanding the above, progress in scaling up FP practices, marked by measures of modern contraceptive prevalence rate (mCPR) and reduction in unmet need at population level, has varied between countries.^12 13^ In addition to the potential role of global policies and programmes, it is important to ascertain examples of low and lower-middle-income countries that have made exceptional progress over the last few decades. Therefore, there is an essential need to examine and gather evidence for country-level FP policies and programmes that have been successful at scale in low-income and middle-income countries.^14^,^17^

This paper will present the methodology used to select exemplar countries representing positive outlier countries that have outperformed expected progress on key FP outcomes, relative to secular trends in human development. Complementary papers cover other aspects of Exemplars such as unpacking the key factors that have contributed to the scale, effectiveness and sustainability of Exemplars in FP in an action-oriented, data-driven way to support decision-makers and drive further advancement in the field of FP.^18^

FP, the focus of this supplement, is one of several critical global health issues investigated under the Exemplars in Global Health (EGH) umbrella. EGH systematically identifies positive outlier countries—those that have achieved exceptional health outcomes despite facing similar resource constraints or contextual challenges as their peers. The core rationale is to rigorously investigate how these countries achieved such disproportionate success, extracting actionable insights and replicable strategies that can inform policy and practice in other low-income and middle-income settings.

Each EGH topic involves a deep dive into country-specific trajectories, leveraging mixed-methods research to understand the complex interplay of policy, programmes and contextual factors driving progress. This current paper, along with others in this supplement, contributes to the FP stream, focusing on specific aspects of FP trajectories to understand pathways to successful FP uptake and continued progress in low-income and low-middle-income countries for global stakeholders.

## Methodology

We know that in contemporary societies, contraceptive use has increased under various conditions. However, contraceptive use has not increased in the absence of an organised FPP. While FPPs have been shown to achieve success in the absence of economic growth (e.g, Bangladesh), impactful results are achieved by countries with secure social settings. The challenge in this methodology is to identify a small set of explanatory variables, predict the expected secular values of the outcome indicators and identify outperforming countries.

We aimed to develop objective metrics and comprehensive criteria for identifying positive outlier countries that have outperformed expected progress relative to predicted values based on some key development indicators (comprising the Human Development Index) on key FP outcomes. The primary outcome indicators analysed were (1) mCPR and (2) demand satisfied for modern methods of contraception among women of reproductive age (15–49 years).

### Approach to analysis

To identify countries with continued progress in FP, the initial round of analysis focused on the period from 2010 to 2020. This timeframe encompasses the resurgence of global interest in FP, marked by the London Summit on Family Planning in 2012, the MDGs and the subsequent global FP2020 movement. By examining outcomes during this period, including mCPR and demand satisfied levels in 2020 and recent changes since 2015, the analysis captures both changes and advancements made following the FP2012 summit.

The overall methodology used for FP Exemplar country selection consisted of linear regressions using one of the FP measures (mCPR or demand satisfied) as the outcome variable and Human Development Index (HDI) as the independent variables. We ran regressions using R (V.4.2.3 and V.4.5.1) and Stata (V.17.0) to investigate the relationship between our FP outcomes and HDI in 2020 to assess the recent levels of mCPR and demand satisfied. We also ran regressions analysing the absolute change in mCPR and demand satisfied in each country and the baseline HDI. We replicated the change regressions for 5-year and 10-year intervals in our study period.

### Country selection rounds

**Figure 1:**
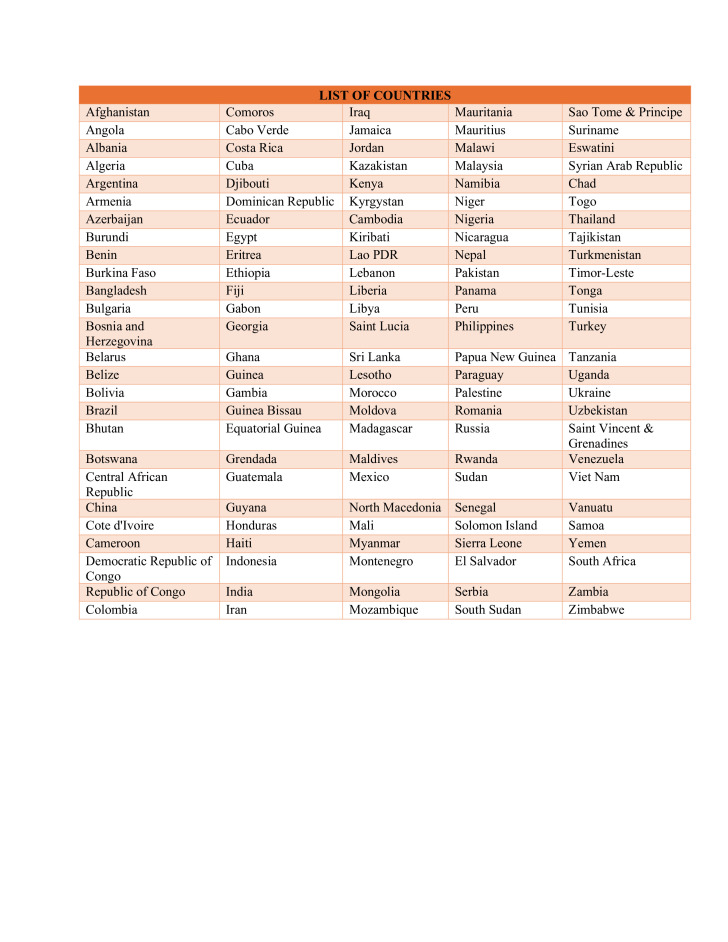
List of countries considered for selection

Simple linear regression was implemented with different outcomes including mCPR, demand satisfied (DS) and change in these two outcomes since 2010 and HDI as the predictor variable (across different time periods as shown in the online supplemental file 1). The countries that deviated from the predicted values of contraceptive prevalence and demand satisfied at a level of significance of 5% and 30% were considered exemplars in FP. Modelled annual estimates of mCPR and demand satisfied data were sourced from UN Population Division and HDI data from 2015 was sourced from UN Development Programme.^8 18^ The final analysis included 130 countries with data available on mCPR, demand satisfied and HDI (figure 1).

For the second round of country selection, we relaxed our residual significance level. We included changes in mCPR and demand satisfied and analysed the same list of countries as for the first round. This was intentional to capture countries that had experienced success in rising contraceptive prevalence beyond sub-Saharan Africa. The relaxation of the earlier selection criteria was able to provide greater geographical spread and regional representation to enhance the project’s depth and relevance in identifying countries beyond just one region and to include countries from other regions such as Latin America and Southeast Asia. Therefore, a 30% level of significance was used, that is, (Observed-Expected, O-E) was greater than (O-E70; Expected at 70% CI), to diversify the country selection pool. Moreover, we initially expanded our timeline to 1994 to capture earlier post-ICPD progress in FP; however, there was no change in the results on country selection.

Total change over a decade (2010–2020) was estimated by breaking it down into two distinct time periods. This approach aimed to maintain a time-lag analysis while assessing the overall change. The total observed change (2010–2020) was calculated by summing the observed changes from the two subperiods, 2010–2015 and 2015–2020. The methodology then introduced three separate estimations of the total change, each based on different statistical significance levels: a general estimated change (E), an estimated change at a 70% significance level (E70), and one at a 95% significance level (E95).

For each round, countries were grouped by region and ranked based on the number of analyses where residuals significantly exceeded expectations according to HDI levels. A composite score was calculated by adding the number of significant outcomes across analysis rounds. Exemplar countries were finally selected from among the highest scoring countries, based on predetermined eligibility criteria. Countries with (1) granularity for subnational comparisons of data sources (DHS), FP services (based on a systematic look at data availability) and (2) credibility and strength of in-country research partners were included. Those countries with (1) a population of less than 5 million, (2) adoption of coercive FP policies (defined as FP law or policy which restricts an individual’s right to establish a family and to decide freely and responsibly on the number and spacing of their children^13^) and (3) classified as conflict settings or presenting safety concerns, were excluded.

Hence, the methodology used for FP exemplar country selection included two important attributes. First, it used a time lag approach to build in an element of causation.^19^ Second, the HDI measure from the start of the time period was used to predict levels of mCPR and demand satisfied. While gross domestic product (GDP) per capita income change has been used most widely in previous exemplar studies, we selected HDI given that it included several elements already known to influence contraceptive adoption.^20^,^23^

## Results

A significant linear relationship between modern contraceptive rate (mCPR2020) and (HDI2019) was observed (F-statistic=42.40, df=1128, p<0.001). Data from 2010 to 2015 for mCPR and demand satisfied for the time period plotted against HDI 2015, showed Malawi, Kenya, Senegal and Sierra Leone to fall on the upper limit of the predicted values range. For data from 2010 to 2020, change in mCPR (2010–2020) with significant residuals above 95% was seen in Malawi, Kenya, Sierra Leone and Senegal. With the same levels of significance, residuals of 95%, total change in demand satisfied (2010–2020) was observed only in Senegal and Sierra Leone.

As shown in table 1, other countries lying on or above the upper limit (95% CI) included Malawi, Lesotho, Cuba and China (marked as ‘x’). Malawi and Lesotho were also placed on or above the upper limit (95% CI) for demand satisfied by modern methods (2020). Moreover, for the time period 2015–2020 for the selected outcome indicators with HDI 2015 as the control metric, countries lying on or above the upper limit (95% CI) included Uganda, Senegal and Peru. Uganda and Senegal were again observed to be placed on or above the upper limit (95% CI) for recent change in demand satisfied by modern methods (2015–2020), with the addition of a few more countries as shown in table 1.

**Table 1:** First round of country selection at 5% significance level for all outcome variables

Country	Level 2020		Recent change 2015–2020		Earlier change 2010–2015		Total change 2010–2020		Number of significant outcomes
	mCPR	Demand satisfied	mCPR	Demand satisfied	mCPR	Demand satisfied	mCPR	Demand satisfied	
Sub-Saharan Africa									
Senegal			x	x		x	x	x	5
Malawi	x	x			x	x	x		5
Sierra Leone					x	x	x	x	4
Lesotho	x	x			x		x		4
Uganda			x	x			x		3
Kenya					x	x	x		3
Latin America									
Peru				x					1
Bosnia			x						1
Cuba	x								1
Bulgaria			x						1
Romania									0
Asia									
China				x					1
Lao People's Democratic Republic									0
Cambodia									0
Timor Leste									0
Malaysia									0

mCPR, modern contraceptive prevalence rate.

It is important to note that most Exemplar countries belong to sub-Saharan Africa, with Lesotho surrounded by South Africa. It is clear that the latest round of change in the uptake of contraception post the 2012 Summit was in/near the African region. Based on the results, the inclusion of Senegal and Malawi as exemplar countries was clear, as both had the greatest number of significant outcomes, as shown in table 1. Following this, Sierra Leone and Lesotho had four significant outcomes each, but Lesotho was excluded based on having a population less than 5 million (as shown in table 2). Furthermore, Sierra Leone was not considered for Round 1 to ensure inclusion of countries from different regions of Africa; however, it was included in Round 2. Bosnia, Cuba and Peru were also excluded based on the least number of significant outcomes and additionally a population of less than 5 million in Bosnia. China from the Asian region was excluded based on its coercive FP policies alongside Lao People's Democratic Republic, Cambodia, Timor-Leste and Malaysia based on no significant outcomes across all time periods (at 5% level of significance) (as outlined in table 2).

**Table 2:** Countries plotted against eligibility criteria

Country	Number of significant outcomes (all time periods across mCPR and demand satisfied)	Coercive policy	Population Less than 5 million
Senegal	5		
Malawi	5		
Kenya	3		
Sierra Leone	4		
Lesotho	4		x
Uganda	3		
Peru	1		
Bosnia	1		x
Cuba	1		
Bulgaria	1		
Romania	0		
China	1	x	
Lao People's Democratic Republic	0		x
Cambodia	0		
Timor Leste	0		
Malaysia	0		

mCPR, modern contraceptive prevalence rate.

Moreover, as shown in table 2, with Senegal in Western Africa and Malawi in Southern Africa, the inclusion of Eastern Africa was considered to ensure a more robust analysis. With Kenya and Uganda, both from Eastern Africa and with a composite score of 3, Kenya was selected over Uganda. The rationale for including Kenya was that the country had an mCPR of 43% for all women and 58% for currently married women versus Uganda at 30% for all women and 39% for currently married women, making it a better fit for selection. Hence, our first three countries for round 1 were Senegal, Malawi and Kenya.

The selection process for round 2 was based on the modified methodology of a 70% cut-off. Additionally, the results were classified based on regions (Latin America, Asia and Africa) to ensure selection of one country from each of these major regions for greater diversity. Within the analysis for outcome indicators for 2020 against HDI, Colombia, Brazil and Cuba were placed on or above the upper limit (70% CI) for both mCPR and demand satisfied as shown in table 2.

For the time period 2015–2020, a shift was seen as none of the countries from the previous period were placed on or above the 70% CI. For both mCPR and demand satisfied, Bolivia was observed to be on or above the 70% CI and was again observed as a positive outlier for both outcomes across 2010–2015, 2010–2020. On the other hand, Colombia was placed on or above the 70% CI for mCPR for 2010–2015, 2010–2020.

While the initial focus on three African Exemplar countries in round one provided valuable insights, it possibly could have limited the potential for additional learning and the identification of broader best practices for other regions. Countries from Asian and Latin American regions were therefore proposed to diversify learning models and capture a broader spectrum of perspectives on Exemplars in FP models. Based on the highest composite score, Lao People's Democratic Republic with a score of four was selected among the top five Asian countries, and Bolivia with a score of six was selected among the top five Latin American countries. Bolivia had the highest number of significant outcomes, that is, a composite score of 6, followed closely by Colombia (score of 3), and Ecuador and Paraguay (score of 1 each). Sierra Leone was also selected for round 2 (with a score of 4) from the top five African countries (shown in table 3). Hence, the countries selected for round 2 were Sierra Leone, Lao People's Democratic Republic and Bolivia. Cuba was excluded based on coercive policies, and Paraguay, Argentina and Ecuador were excluded based on having the lowest scores (least number of significant outcomes in the region), as shown in table 4.

**Table 3:** Second round of country selection at 30% significance level for all outcome variables

Country	Level 2020		Recent change 2015–2020		Earlier change 2010–2015		Total change 2010–2020		Number of significant outcomes
	mCPR	Demand satisfied	mCPR	Demand satisfied	mCPR	Demand satisfied	mCPR	Demand satisfied	
Latin America									
Bolivia			x	x	x	x	x	x	6
Colombia	x	x			x				3
Paraguay	x								1
Argentina					x				1
Ecuador	x								1
Asia									
Lao People's Democratic Republic					x	x	x	x	4
Bangladesh	x	x							2
Myanmar		x							1
China	x	x							2
Sub-Saharan Africa									
Sierra Leone					x	x	x	x	4
Lesotho	x	x			x		x		4
Burkina Faso			x	x			x		3
Republic of Congo				x					1
Uganda			x	x			x		3

mCPR, modern contraceptive prevalence rate.

**Table 4:** Countries plotted against eligibility criteria (for second round of country selection)

Country	Number of significant outcomes (all time periods across mCPR and demand satisfied)	Coercive policy	Population less than 5 million
Bolivia	10		
Colombia	6		
Paraguay	5		
Argentina	5		
Ecuador	5		
Lao People's Democratic Republic	8		
Bangladesh	3		
Myanmar	3	x	
China	2	x	
Thailand	2		
Sierra Leone	6		
Lesotho	8		x
Burkina Faso	5	x	
Republic of Congo	5		x
Uganda	4		

mCPR, modern contraceptive prevalence rate.

Thus the final countries selected included Senegal, Malawi and Kenya for the first round and Sierra Leone, Lao People's Democratic Republic and Bolivia for the second round.

## Discussion

In 2015, the United Nations Sustainable Development Goals (SDGs) succeeded the MDGs as the new global development framework, guiding progress until 2030. The SDGs, unlike their predecessors, explicitly include Sexual and Reproductive Health and Rights, a significant achievement attributed to the persistent advocacy of women’s health activists.^1 14^ It is argued that the MDGs, with their narrow focus, may have inadvertently reduced women’s health to a limited set of concerns.^14 24^ In contrast, the SDGs are viewed as an opportunity to comprehensively address the broader women’s health agenda.^2^ Both the MDGs and SDGs encompass targets related to maternal and child health and gender equality, crucial determinants for successful FPPs.

Despite this emphasis on FP, progress at the country level has been uneven. Of the over 90 partners involved in FP2020, only 38 countries have improved and expanded access to voluntary, rights-based, high-quality FP services, while 11 countries have achieved or are on track to achieve their FP2020 goals.^2 25^ This highlights the critical need to study successful large-scale FP initiatives in various regions. To address this, we developed a methodology to identify countries with accelerated progress in FP. Our initial selection included Kenya, Malawi and Senegal, representing diverse regions within Africa. The second round expanded this to include Lao People's Democratic Republic, Bolivia and Sierra Leone, further diversifying regional representation to encompass Asia and Latin America alongside Africa.

The African region serves as a valuable exemplar in FP. A systematic review by Mwaikambo *et al* analysed successful FP interventions between 1995 and 2008.^26^ Among the 63 studies reviewed, 73% reported positive outcomes in Africa. The most successful FP achievements appear (in the literature) to be in the early 2000s in Sierra Leone, Ethiopia and Kenya.^26^,^29^ However, while some sub-Saharan African countries such as Kenya and Malawi have made significant strides towards increasing contraceptive access and coverage in a short period of time, others such as Nigeria and the Democratic Republic of Congo (DRC) continue to lag.^16 28^ This may be due to a lack of donor support, internal conflicts, despite their respective governments’ bold pledges at the Summit.^29^

Moreover, the integration of FP services within existing healthcare systems, particularly in Southeastern Asia during the 1980s, has contributed to increased uptake of FP services. Lao People's Democratic Republic and Vietnam exemplify this approach, showcasing improved accessibility for women and young girls visiting healthcare facilities and utilizing contraception.^30^ Therefore, the selection of an exemplar country within this region can offer valuable insights into the regional impact of FP achievements and continued progress.^31^

Bolivia also serves as an important country-level case study of successful FPPs that are not state-led within the Latin American region. The country has had a continued decline in fertility rates over the past three decades. It stands out by prioritising the sexual and reproductive health needs of adolescents, recognising FP as an integral component of broader societal well-being.^32^ However, other countries in the region, like Colombia and Honduras, despite some progress since the 1990s, haven’t witnessed the same significant declines in unmet need observed in other regions.^33^ This supports the selection of Bolivia as an exemplar country as it remains a positive outlier in the region. The learnings from the country’s journey can provide relevant and valuable insights into FP initiatives that are acceptable, appropriate and scalable specifically in the Latin American context.

To identify these countries with continued success in FP over a longer period of a decade rather than short spurts, the initial round focused on the 2010–2020 period.^16 34^ This timeframe encompasses a period of renewed global emphasis on FP, characterised by key events such as the 2012 London Summit on Family Planning, the ongoing influence of the MDGs/SDGs and the subsequent launch of the global FP2020 initiative.^1635^,^37^

Furthermore, while traditional methods can contribute to reducing population fertility rates, studies emphasise the importance of focusing on modern methods of contraception due to their higher individual effectiveness.^38 39^

This study is uniquely different from previous exemplar studies in which GDP has traditionally been used to select exemplar countries^40^, as it uses the HDI from a previous period for selection. The HDI is composed of three dimensions reflecting life expectancy, education (mean and expected years of schooling), and standard of living (gross national income per capita). These are established as conducive to an increase in demand satisfied and mCPR.^40^ In the past, exemplar selection for other topics generally relied on change in comparison with GDP per capita and annual economic growth as undertaken through the sprint analysis for FP by Gates Ventures.^40^ However, based on the literature and extensive analysis using income measures, it is clear that GDP change alone was not a strong predictor of contraceptive prevalence.^41 42^ Moreover, the HDI is a composite index that goes beyond GDP to include additional elements of education and life expectancy and hence has the advantage over using these indicators individually.^42^ The combined application of (mCPR), demand satisfied and HDI metrics, in this study, strengthens the face validity of the current methodology. Face validity, as defined by Nunnally,^43^ refers to the extent to which a measure appears to assess its intended construct.

### Limitations

While the HDI provides an important metric for country selection, its composite nature makes it difficult to discern the specific impact of each of its individual components on FP. This is particularly relevant in undertaking in-depth country studies, in which isolating the effects of FP policies, strategies and interventions on each HDI component becomes complex and challenging.

Moreover, data availability presents a significant challenge. In particular, the measure of women’s rights and empowerment as supported at the state-level policy and also in societal terms, was lacking. With available data mostly prioritising national statistics, in-country and regional variations that are crucial to understanding the context of FP and contraceptive uptake are missed. This masks important disparities in access to and use of FP services and uptake among various regions, ethnicities or socioeconomic groups.

The reliability of available data is further affected by reporting biases, missing points and the limitations specific to methods of data collection. Besides these, the greater challenge remains limited access to recent data, particularly in the case of disaggregated subnational indicators, which impedes a more holistic approach to country selection.

A deeper historical perspective, such as that offered in The Global Family Planning Revolution (2007), highlights numerous countries not identified as exemplars in current analyses but which nevertheless achieved significant FP milestones. Nations like Indonesia, Thailand, Bangladesh, Turkey, Jamaica, Chile and Egypt demonstrate a rich history of successful FPP implementation, reaching impressive mCPR levels in the late 1990s. These nations, having transitioned away from receiving aid that included survey support, no longer have their FP trajectories consistently captured by Demographic and Health Survey (DHS) data. Thus, the reliance on DHS and the Multiple Indicators Cluster Survey (MICS) data inherently creates an intrinsic bias in country coverage.

### Future directions

Future research on selection of exemplar countries in FP requires a deeper focus on equity, empowerment and informed choice. Based on previous exemplar studies evidence from the Countdown for 2015 and 2030 project, equity has several dimensions, with wealth or asset quintiles representing just one, which when applied, would limit the pool of countries for selection due to data inconsistencies and lack of universal availability.^38^ In a recent review conducted by the partnership on High Impact Practices in Family Planning, or HIPs, the selection of countries on the basis of one sole form of equity measures (e.g, the level or rate of change of mCPR coverage in the poorest quintile) may not adequately capture other important measures of equitable access represented by factors such as residence, geography, ethnicity^41 44^—all of which constitute emerging and important measures of disparities.

Moreover, while surveys and studies exploring the drivers of change on empowerment—such as personal agency and voluntary contraceptive use—are a recent development in the research landscape, this study establishes a set of exemplar countries. Moving forward, we will conduct individual case studies in each selected country. These case studies will delve into different dimensions of women’s rights and autonomy, particularly focusing on (1) decision-making autonomy in household affairs and how much control women have over decisions impacting their lives and (2) physical mobility and freedom of movement and how this influences access to FP information, services and overall sexual and reproductive health outcomes.

Additionally, information on method mix as well as the method information index (MII)—which assesses the quality of family counselling services and measures the extent to which service providers inform clients about the ‘best fit’ for contraceptive options, side effects and related management—has been identified as a critical measure of quality of services by choice.^45^ However, data for the MII is currently available for only 42 countries (Track 20). Therefore, the deep dive, rather than the country selection process, is better suited to exploring informed choice at the country and subnational levels (given that further country-specific data exploration will be possible in that phase).

## Conclusions

Despite global commitment to expand FP access, progress varies greatly. While some countries have seen impressive gains, others continue to struggle. Our unique approach departed from other exemplar studies done previously, to assess not just economic factors, but also crucial dimensions like life expectancy and education relative to FP outcomes. We feel this is an important advancement that we recommend for future exemplar selection.

Carefully considering time periods and ensuring regional representation, the final set of selected countries will serve as the foundation for the next phase of research. This phase will dive deeper into the unique FP trajectories, strategies and lessons learnt at country level and explore the drivers of change. This will provide valuable evidence-based strategies for replication and scaling up successful interventions in other countries within the same regions that are experiencing slower progress.

Moving forward, this study proposes further exploring equity, empowerment and informed choice. Doing so will provide a deeper understanding of the factors driving progress in exemplar countries, offering valuable insights to improve FP and establish related high impact practices at the country level, regional level and gradually global level.

## Reflexivity statement

This research paper is a collaborative effort reflecting the work of a consortium comprising members from The Aga Khan University (AKU), Population Council, SickKids (The Hospital for Sick Children) and the Exemplars in Global Health (EGH) team. The consortium possesses extensive and long-standing experience in the field of FP, deeply believing in its critical role as a determinant of improved maternal and child health outcomes. The EGH team brings prior experience from the overarching Exemplars in Global Health study, which focuses on identifying and analysing successful countries and their respective trajectories in various global health domains.

The consortium maintains an established history and advocacy within the FP sector. Specifically, the EGH team’s previous work on ‘exemplar’ countries in FP within low-income and lower-middle-income countries has shaped our investigative lens, leading us to examine and identify replicable successes. This focus on low-income and lower-middle-income countries necessitated the development of a novel methodological approach, distinct from earlier EGH studies, to encompass indicators beyond conventional economic metrics like GDP.

As a consortium, we collectively operate under the fundamental assumption that the adoption and success of FP, particularly modern contraceptive practices, are profoundly influenced by each country’s unique political, social and economic landscape. Throughout the entire project, we have systematically explored these influences using a variety of qualitative and quantitative methodologies (detailed statement attached as online supplemental material 1).

## Supplementary material

10.1136/bmjgh-2024-018771 online supplemental file 1

10.1136/bmjgh-2024-018771 online supplemental material 1

## Data Availability

Data are available on reasonable request.
