# Supplementary material for: Family planning exemplar country selection methodology: time lag and trends analysis
Source: BMJ Glob Health. 2026 Jun 9;11(Suppl 3):e018771. doi: 10.1136/bmjgh-2024-018771 (PMC13250220; doi:10.1136/bmjgh-2024-018771)
Supplement: online supplemental file 1 [file bmjgh-11-Suppl_3-s001.pdf]

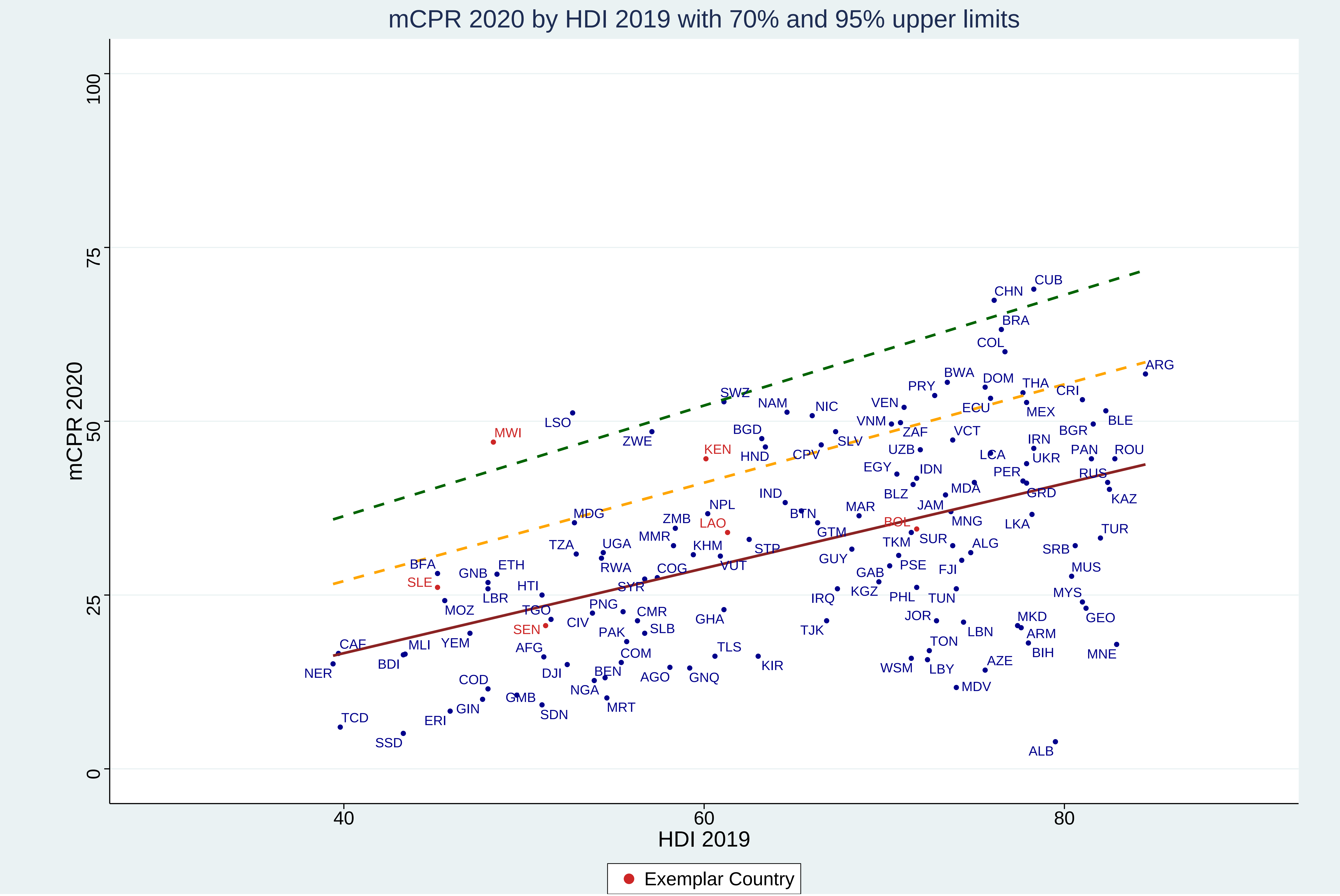

R-squared = 0.2488

y = 0.610*x-7.77

Upper limit 70% CI: y = 0.708*x-1.30

Upper limit 95% CI: y = 0.796*x+4.53

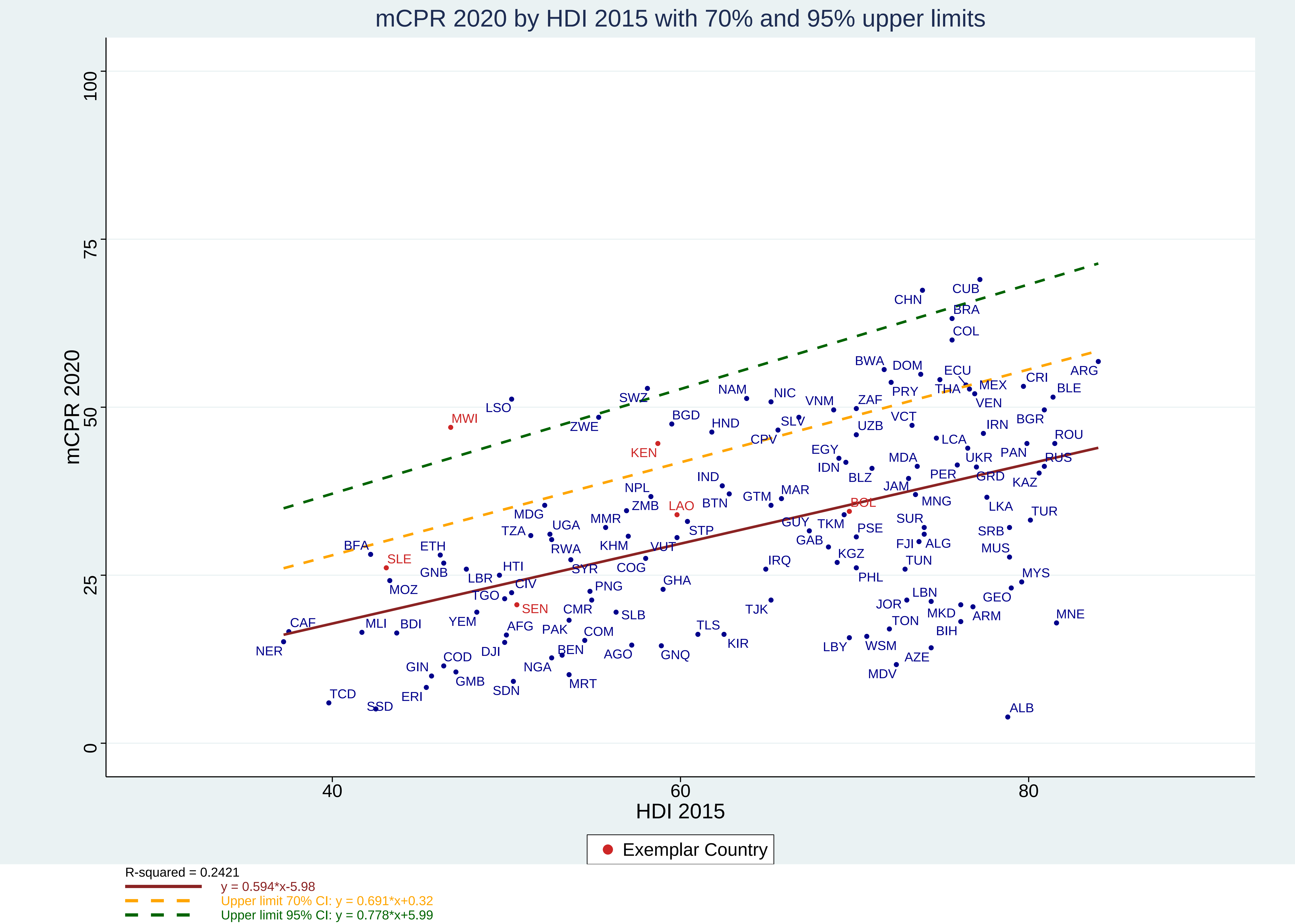
mCPR 2020 by HDI 2015 with 70% and 95% upper limits
mCPR 2020
HDI 2015
0
25
50
75
100
40
60
80
CHN
CUB
BRA
COL
BWA
DOM
ECU
ARG
CRI
THA
MEX
PRY
BLE
VEN
NAM
NIC
VNM
ZAF
SWZ
LSO
MWI
ZWE
BGD
HND
SLV
VCT
UZB
IRN
BGR
LCA
ROU
KEN
CPV
EGY
IDN
MDA
UKR
PAN
RUS
IND
BLZ
PER
GRD
KAZ
NPL
GTM
MAR
JAM
MNG
LKA
ZMB
LAO
BTN
BOL
TUR
MDG
MMR
GUY
TKM
SUR
UGA
STP
PSE
SRB
TZA
GAB
FJI
ALG
BFA
ETH
RWA
KHM
VUT
KGZ
MUS
SLE
TUN
GNB
HTI
SYR
COG
IRQ
LBR
PHL
MYS
MOZ
CIV
PNG
GHA
LBN
TGO
JOR
GEO
SEN
CMR
TJK
MKD
ARM
CAF
SLB
MNE
MLI
BDI
YEM
AFG
TON
PAK
COM
TLS
BIH
KIR
NER
DJI
BEN
LBY
WSM
AGO
GNQ
AZE
COD
GIN
NGA
GMB
MDV
MRT
SDN
TCD
ERI
SSD
ALB
Exemplar Country
R-squared = 0.2421
y = 0.594*x-5.98
Upper limit 70% CI: y = 0.691*x+0.32
Upper limit 95% CI: y = 0.778*x+5.99

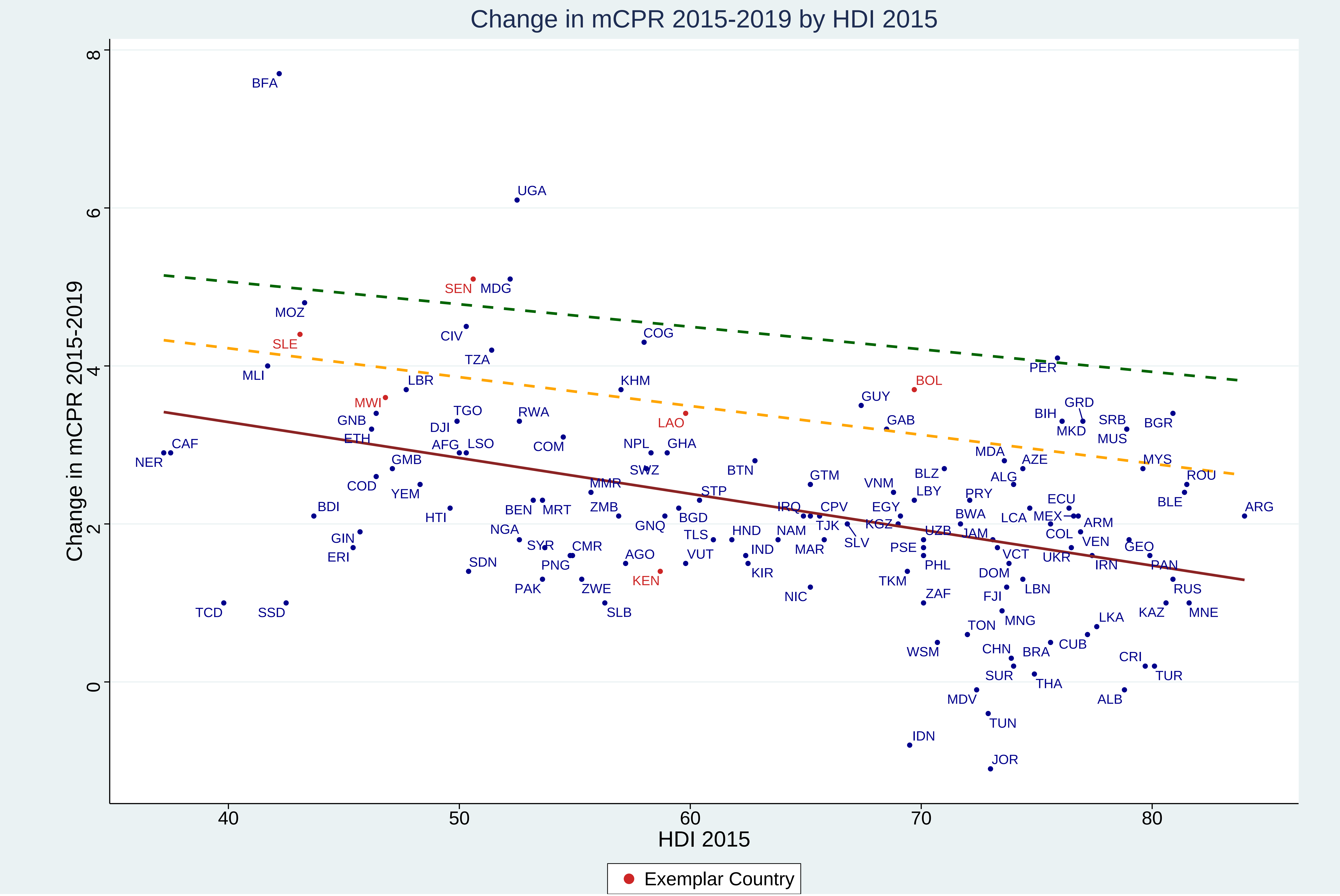

R-squared = 0.1808

y = -0.045*x+5.11

Upper limit 70% CI: y = -0.037*x+5.68

Upper limit 95% CI: y = -0.029*x+6.21

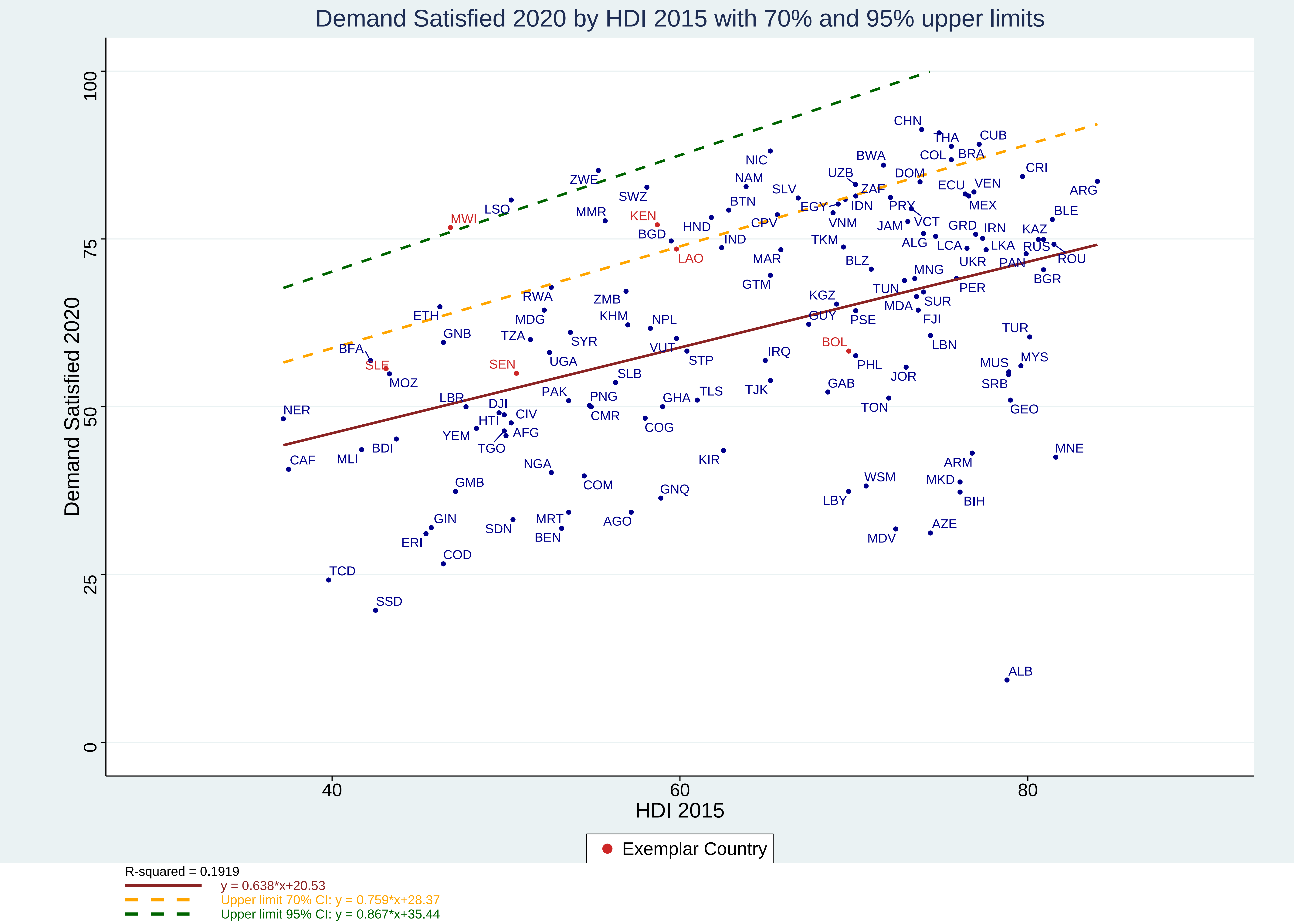

Demand Satisfied 2020 by HDI 2015 with 70% and 95% upper limits
Demand Satisfied 2020
HDI 2015
Exemplar Country
R-squared = 0.1919
y = 0.638*x+20.53
Upper limit 70% CI: y = 0.759*x+28.37
Upper limit 95% CI: y = 0.867*x+35.44

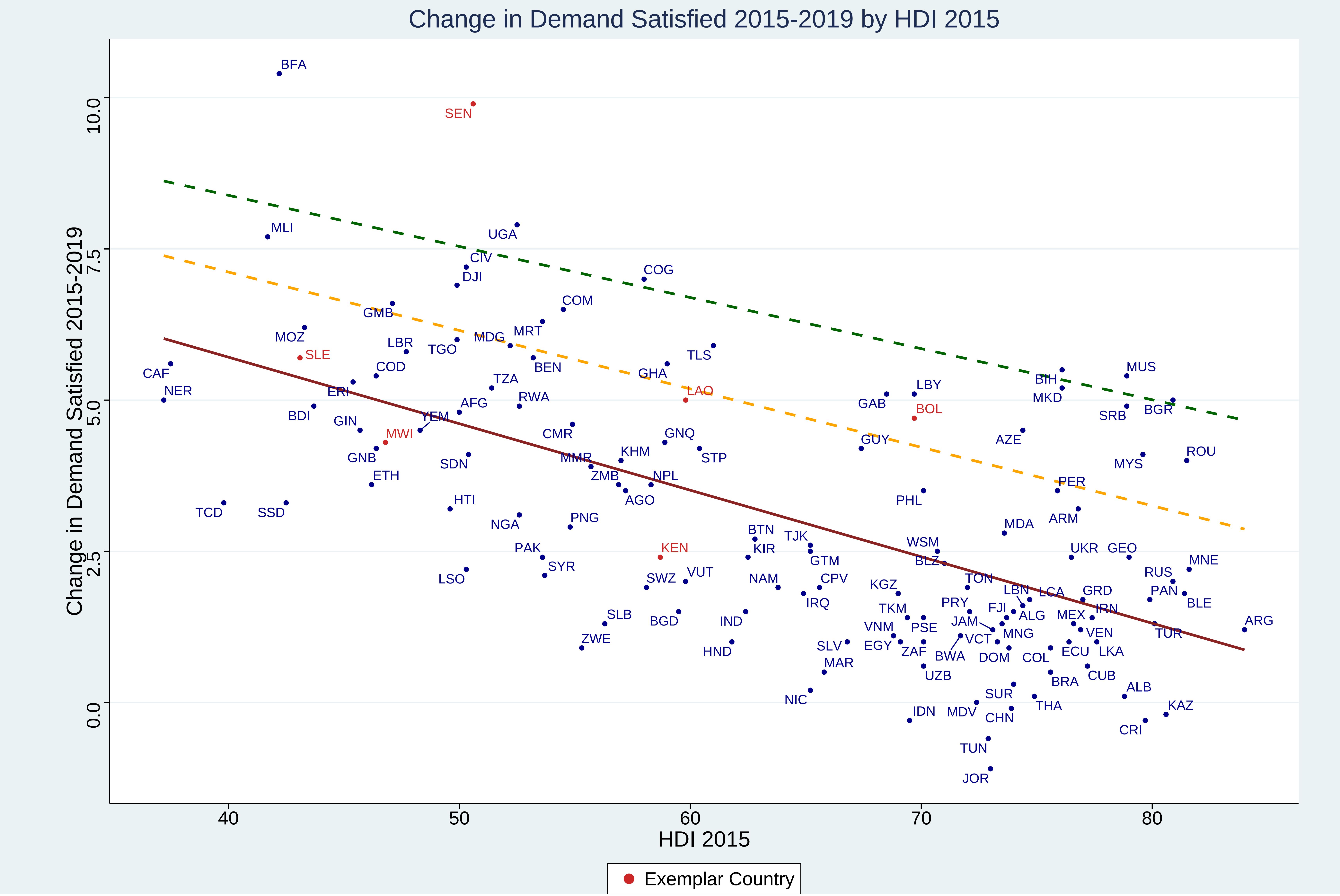

R-squared = 0.3632

y = -0.110*x+10.11

Upper limit 70% CI: y = -0.097*x+10.98

Upper limit 95% CI: y = -0.085*x+11.77
