# Supplementary material for: Family planning exemplar country selection methodology: time lag and trends analysis
Source: BMJ Glob Health. 2026 Jun 9;11(Suppl 3):e018771. doi: 10.1136/bmjgh-2024-018771 (PMC13250220; doi:10.1136/bmjgh-2024-018771)
Supplement: online supplemental material 1 [file bmjgh-11-Suppl_3-s002.pdf]

BMJ Global Health Author Reflexivity Statement

Adapted from Morton, B., Vercueil, A., Masekela, R., Heinz, E., Reimer, L., Saleh, S., Kalinga, C., Seekles, M., Biccard, B., Chakaya, J., Abimbola, S., Obasi, A. and Oriyo, N. (2022), Consensus statement on measures to promote equitable authorship in the publication of research from international partnerships. Anaesthesia, 77: 264-276. https://doi.org/10.1111/anae.15597

Title: Family Planning Exemplar Country Selection Methodology: Time Lag and Trends Analysis (bmjgh-2024-018771)

**Study conceptualisation**	
1. How does this study address local research and policy priorities?	This study was designed to identify positive outlier countries that achieved the accelerated progress in voluntary uptake of the modern methods of contraception and demand satisfied for family planning (FP). FP is an issue of major importance and priority in low- and middle-income countries (LMICs). The project seeks to connect and engage in, through a consortium, collaboration with research experts from both high-income and low-middle income countries to learn the drivers of change and how the solutions could be adapted to similar contexts to design evidence informed policies and program to improve the uptake of FP.
2. How were local researchers involved in study design?	The first category of researchers involved were those with extensive experience of conducting, leading, and organising international research collaborations including the Gates Foundation, AKU, SickKids and Population Council.<br>The second category were in-country researcher partners (emerging from research undertaken as a next step to the selection of countries detailed in this paper) identified for their technical expertise in reproductive health and family planning, experience with large-scale quantitative and qualitative research, and established networks with national stakeholders and government institutions. These included the International Centre for Reproductive Health (Kenya), African Institute for Development Policy (Malawi), Université Cheikh Anta Diop (Senegal), Social Development Solution Network (Bolivia), Lao Tropical and Public Health Institute (Lao PDR), and the Institute of Community and Public Health (Sierra Leone). Based on projective objectives and conceptual framework, we developed specific Terms of Reference and roles for each partner including collaboration with relevant government authorities and applying for ethics approval for research. The local researcher helped in contextualizing the study methods, tools and prioritization based on the in country needs identified by the diverse stakeholders.
**Research management**	
3. How has funding been used to support the local research team(s)?	The overall Exemplars project has been used to support in-country research partners (emerging from research undertaken as a next step to the selection of countries detailed in this paper) by providing them with technical expertise and guidance in research and funding for publications as well as funding for their participation in both national and international forums, including workshops, seminars, and conferences for dissemination of their research.
**Data acquisition and analysis**	
4. How are research staff who conducted data collection acknowledged?	Research staff involved have been included as authors. Each member delivered specific role/s, as discussed and agreed upon earlier, during the writing, review, and finalization process and have been acknowledged accordingly.
5. How have members of the research partnership been provided with access to	All members of the partnership have access to data through an online drive with all data and documents for respective countries.

study data?	
6. How were data used to develop analytical skills within the partnership?	All in-country partners (emerging from research undertaken as a next step to the selection of countries detailed in this paper) were provided guidance and technical assistance in the process of their research components including systematic review, policy, program, and financing review, and qualitative study design. Assistance included technical support in data analysis and write-up for each component.
**Data interpretation**	
7. How have research partners collaborated in interpreting study data?	This was an iterative process. For each stage of the country selection method, including design and respective rounds of selection, the consortium met, reviewed the data, and discussed justification for the selection of the country, as well as concerns and iterative insights on how to address limitations from the first round of selection in the subsequent round.
Drafting and revising for intellectual content	
8. How were research partners supported to develop writing skills?	The research team is composed of senior researchers and academicians. Early-career individuals on the authorship team were supported by these personnel to develop and refine their writing skills based on multiple rounds of draft development, review, and modification.
9. How will research products be shared to address local needs?	We developed a publication dissemination plan in consultation with the consortium and in-country research partners. This plan included engagement with research leaders in global health and international collaborations, for various research component implementation, post-research seminars, conferences, workshops at country, regional and global levels.
**Authorship**	
10. How is the leadership, contribution and ownership of this work by LMIC researchers recognised within the authorship?	Z.S, Z.A.B, M.M, J.K.D and Z.M spearheaded, as senior researchers, the research's conceptualization and were instrumental in the design and execution of the methods. We specifically included researchers based in the Global South, including those from AKU and the Population Council.
11. How have early career researchers across the partnership been included within the authorship team?	The original draft was penned by early-career individuals including A.M and I.M. The draft was subsequently reviewed by all authors. Feedback from them was incorporated by A.M and I.M., and the final version was completed with Z.M, Z.S, Z.A.B, J.K.D providing oversight for the manuscript's overall direction.
12. How has gender balance been addressed within the authorship?	There are more female authors than male authors.
**Training**	
13. How has the project contributed to training of LMIC researchers?	In-country research partners from LMICs (emerging from research undertaken as a next step to the selection of countries detailed in this paper) benefited from technical guidance and expertise across their research activities. Furthermore, we provided funding to support subsequent publications and to ensure their participation in international and national dissemination forums, such as conferences, etc.
**Infrastructure**	
14. How has the project contributed to improvements in local infrastructure?	This project has not directly contributed to improvements in local infrastructure. Rather, all research components, from the countries selected through the methods specified in this paper, will provide a set of recommendations for scaling up family planning to improve contraceptive

	uptake and reduce unmet need in LMICs (that are lagging behind). Moreover, all equipment procured through the grant remains with the local institutions, and in-country research partners have been provided capacity-building opportunities in research methodology and data collection pertaining to the project.
Governance	
15. What safeguarding procedures were used to protect local study participants and researchers?	There was no primary data collection as part of this selection methodology paper; therefore, this question is not directly applicable. The issue of safeguarding for all local in-country research partners (emerging from research undertaken as a next step to the selection of countries detailed in this paper), as applied in their respective research components, has been considered and explained in distinct manuscripts relating to country case studies (proceeding this paper).
